# Analytical Guidelines for Designing Curvature-Induced Dielectrophoretic Particle Manipulation Systems

**DOI:** 10.3390/mi11070707

**Published:** 2020-07-21

**Authors:** Akshay Kale, Amirreza Malekanfard, Xiangchun Xuan

**Affiliations:** 1 Electrical Engineering Division, CAPE Building, Department of Engineering, University of Cambridge, Cambridge CB3 0FA, UK; 2 Department of Mechanical Engineering, Clemson University, Clemson, SC 29634, USA; amaleka@g.clemson.edu (A.M.); xcxuan@clemson.edu (X.X.)

**Keywords:** microfluidics, dielectrophoresis, curvature-induced, electrokinetic, particle focusing

## Abstract

Curvature-induced dielectrophoresis (C-iDEP) is an established method of applying electrical energy gradients across curved microchannels to obtain a label-free manipulation of particles and cells. This method offers several advantages over the other DEP-based methods, such as increased chip area utilisation, simple fabrication, reduced susceptibility to Joule heating and reduced risk of electrolysis in the active region. Although C-iDEP systems have been extensively demonstrated to achieve focusing and separation of particles, a detailed mathematical analysis of the particle dynamics has not been reported yet. This work computationally confirms a fully analytical dimensionless study of the electric field-induced particle motion inside a circular arc microchannel, the simplest design of a C-iDEP system. Specifically, the analysis reveals that the design of a circular arc microchannel geometry for manipulating particles using an applied voltage is fully determined by three dimensionless parameters. Simple equations are established and numerically confirmed to predict the mutual relationships of the parameters for a comprehensive range of their practically relevant values, while ensuring design for safety. This work aims to serve as a starting point for microfluidics engineers and researchers to have a simple calculator-based guideline to develop C-iDEP particle manipulation systems specific to their applications.

## 1. Introduction

Obtaining a pre-concentrated sample of particles is an important step for its use in subsequent operations in micro total analysis systems (µTAS) [1,2,3,4]. The particle concentration process in these microfluidics-based systems is commonly brought about by manipulation of particle motion inside the “active regions” located in microfluidic flows with the help of an external energy field-induced force in conjunction with the hydrodynamic drag force. Such manipulation leads to a deflection of the particle motion from its unperturbed direction, which is induced by the component of the external force orthogonal to the flow direction. The resulting motion of the particles causes them to flow through a more confined volume within the microfluidic channel, thereby focusing them and increasing their local concentration [5]. This concentrated sample can then be extracted for further downstream processing by designing an outlet junction to the primary channel and exploiting the laminar nature of microfluidic flows. The particle concentration forces induced by the energy fields are functions of the intrinsic properties of the particles relative to those of the fluid. Hence, no physical labels need to be attached to the particles for tagging them. Since these tags can affect the particle phenotype, especially in the case of biological particles, the concentration process are entirely non-invasive to the particles [6]. This non-invasive nature of the concentration process makes it particularly suitable to biomedical research where retaining the phenotype of the bio-particle is extremely crucial. Depending upon the type of external energy field applied, the concentration processes can be induced acoustically [7,8], magnetically [9,10], optically [11,12], hydrodynamically [13,14], or dielectrophoretically [15]. Along with the advantages of a non-invasive nature and a cubic scaling of the concentration force with particle size [16], which are offered by each of the aforementioned methods, dielectrophoretic particle concentration offers additional benefits: (A) easier integration with the subsequent downstream electrically driven detection systems [17] and (B) possibility to exploit the plug-like velocity profile of the bulk electroosmotic fluid flow for transporting the particles, which avoids dispersion issues prevalent in pressure driven flows [18].

The gradients of the external energy field in dielectrophoresis (DEP) are of an electrical nature, and hence, the dielectrophoretic concentration of particles occurs due to the differences in the electrical properties of the particles relative to the fluid [19,20,21]. The dielectrophoretic force acting on a polarisable sphere with a diameter *d*, an electrical conductivity $\sigma_{p}$ and a dielectric constant $\varepsilon_{p}$ inside a fluid with an electrical conductivity $\sigma_{f}$ and a dielectric constant $\varepsilon_{f}$ can be expressed as follows:

(1) $$F_{\mathrm{DEP}}=\frac{\pi }{4}d^{3}\varepsilon_{f}Real\left(f_{CM}\right)\nabla \left(E\cdot E\right)\;\mathrm{where}\;f_{CM}=\left[\frac{\left(\varepsilon_{p}-\varepsilon_{f}\right)+j\frac{\left(\sigma_{p}-\sigma_{f}\right)}{2\pi f}}{\left(\varepsilon_{p}+2\varepsilon_{f}\right)+j\frac{\left(\sigma_{p}+2\sigma_{f}\right)}{2\pi f}}\right];\;j=\sqrt{-1}$$

where **E** is the externally applied electric field, *f* is the frequency of the applied voltage and $f_{CM}$ is the Clausius-Mossotti factor of the particle-fluid system. Based on the method of inducing electrical energy gradients, i.e., ∇(E·E), dielectrophoretic concentration process can be broadly classified as electrode-based (i.e., eDEP, where the gradients are generated by a set of patterned [22,23,24,25,26,27,28] or virtual electrodes [29]), insulator-based (i.e., iDEP, where the gradients are generated by non-uniform cross sections within the microfluidic circuit [30,31,32,33,34,35]), or curvature-induced (i.e., C-iDEP, where the curvature of the microfluidic channel produces unequal electric field intensities across a channel cross section [36]). Simply making the channel curved is sufficient to generate DEP, and hence, the cross section of the microfluidic channel need not be reduced, thereby rendering C-iDEP systems much less susceptible to localised Joule heating effects that are more prevalent in their insulator-based counterparts [37,38,39,40]. Similarly, the typical fluid-reservoir-based electrode insertion outside the flow channel not only renders the fabrication simple, but also keeps these systems relatively safe from electrolysis compared to the metallic microelectrode-based eDEP. Additionally, the curvature facilitates the use of a longer channel length within a given area compared to a straight channel, and this greatly increases the area utilisation of lab-on-a-chip systems implementing C-iDEP [41].

While several reports in the existing literature have demonstrated the use of C-iDEP for manipulating particles and cells [42,43,44,45,46,47], a rigorous mathematical treatment of the dielectrophoretic particle dynamics for these systems is still lacking. Such a treatment will prove to be extremely helpful in providing guidelines to design C-iDEP particle manipulation systems for lab-on-a-chip applications. This work demonstrates a fully analytical treatment of the underlying physics of C-iDEP. Briefly, a circular arc microchannel, which represents the most basic design for C-iDEP particle manipulation systems, is considered for the analysis. Elegant, fully dimensionless equations of the pathline of a particle undergoing DEP in this channel geometry are established from first principles. It is shown that the particle dynamics and the design of circular arc microchannels can be fully controlled by three dimensionless parameters. Two-dimensional finite element simulations are used to support the analysis and are shown to agree with the theoretical equations with great accuracy. This work establishes a simple calculator-based approach to enable microfluidics engineers and scientists to design C-iDEP systems with a factor of safety.

## 2. Theory and Analysis

### 2.1. Dielectrophoretic Particle Dynamics in a Circular Arc Microchannel 

**Figure 1:**
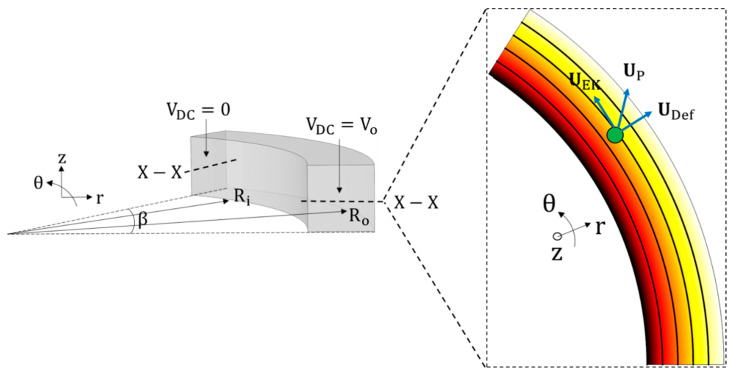
Schematic of a circular arc microchannel explaining the dimensions and the r-θ-z coordinate system, and framework used for a theoretical analysis of the underlying physics of curvature-induced dielectrophoresis (C-iDEP). The 2D projection of the r-θ section plane shows the components of the particle velocity, $u_{P}$, inside the microchannel, consisting of the stream-wise electrokinetic ($u_{\mathrm{EK}}$) component and the cross-stream dielectrophoretic deflection ($u_{\mathrm{Def}}$) component. The electric field contour (the darker colour the larger magnitude) and lines (equivalent to the fluid streamlines [48]) are superimposed to explain the orientation of the velocity components relative to the electric field. A situation of negative DEP is shown, where the particle will move from the inner channel wall to the outer wall. For a positive DEP, the resultant velocity and the deflection component will reverse their directions. Note that the underlying physics of C-iDEP are two dimensional because of the application of the voltage over the entire r-z plane.

Figure 1 describes a generalised geometry for the theoretical analysis of particle physics in C-iDEP systems. Let $V_{0}$ be a DC voltage drop biased with an AC voltage characterised by an RMS AC to DC ratio α applied across a circular arc microfluidic channel. The channel has a uniform width *W*, a uniform height *H* and a mean radius of curvature *R*_c_. The arc subtends an angle β at the centre of the curvature of the channel. Assuming (a) a thin electric double layer limit, (b) negligible displacement currents and (c) uniform liquid properties, the applied DC voltage drop generates an electric field $E_{\mathrm{DC}}$ inside the microchannel, which is governed by the Laplace equation. In cylindrical coordinates, this can be expressed as

(2) $$\frac{1}{r}\frac{\partial }{\partial r}\left(r\frac{\partial V_{\mathrm{DC}}}{\partial r}\right)+\frac{1}{r^{2}}\frac{\partial^{2}V_{\mathrm{DC}}}{\partial \theta^{2}}+\frac{\partial^{2}V_{\mathrm{DC}}}{\partial z^{2}}=0$$

(3) $$E_{\mathrm{DC}}=-\nabla V_{\mathrm{DC}}=-\frac{\partial V_{\mathrm{DC}}}{\partial r}\hat{r}-\frac{\partial V_{\mathrm{DC}}}{r\partial \theta }\hat{\theta }-\frac{\partial V_{\mathrm{DC}}}{\partial z}\hat{z}$$

where $V_{\mathrm{DC}}$ is the DC potential field. $\hat{r}$, $\hat{\theta }$ and $\hat{z}$ represent the unit vectors in the radially outward direction, the counter-clockwise angular direction and the upward direction along the channel height respectively. Recognising that the voltage drop is applied across the entire cross-section of the channel represented by the r-z plane, the above equations simplify to an ordinary differential equation expressed as

(4) $$\frac{d^{2}V_{\mathrm{DC}}}{d\theta^{2}}=0;\;E_{\mathrm{DC}}=-\frac{1}{r}\frac{dV_{\mathrm{DC}}}{d\theta }\hat{\theta };\;\mathrm{subject}\;\mathrm{to}\;V_{\mathrm{DC}}\left(\theta =0\right)=V_{0};\;V_{\mathrm{DC}}\left(\theta =\beta \right)=0$$

The solution of Equation (4) is given as

(5) $$V_{\mathrm{DC}}=V_{0}\left(1-\frac{\theta }{\beta }\right);\;E_{\mathrm{DC}}=\frac{V_{0}}{\beta r}\hat{\theta }$$

Due to this electric field, a spherical particle inside the microchannel containing a liquid of dynamic viscosity $\eta_{f}$ experiences an electrokinetic velocity $u_{\mathrm{EK}}$, which can be written as

(6) $$u_{\mathrm{EK}}=\frac{\varepsilon_{f}\left(\zeta_{p}-\zeta_{w}\right)E_{\mathrm{DC}}}{\eta_{f}}=\frac{\varepsilon_{f}\left(\zeta_{p}-\zeta_{w}\right)V_{0}}{r\beta \eta_{f}}\hat{\theta }$$

where $\zeta_{p}$ and $\zeta_{w}$ represent the particle and wall zeta potentials respectively. For negative wall and particle zeta potentials (which is usually valid for microfluidic particle handling systems [49]), the electrokinetic motion occurs in the positive (counter-clockwise) direction along the arc length if the wall zeta potential is lower than the particle zeta potential (i.e., $\zeta_{w}<\zeta_{p}$) and in the negative (clockwise) direction if vice versa. Note that the AC voltage does not contribute to the electrokinetic motion of the particles because of the linear dependence of the electrokinetic velocity with the electric field (which causes the time average of the AC electric field over a cycle to vanish).

It is also observed from Equation (5) that the electric field inside the microchannel is a function of the radial co-ordinate, thereby making it non-uniform. The resulting electric field gradients inside the channel can be expressed using the expression for DC electric field (Equation (5)) and the definition of the gradient vector in cylindrical co-ordinates (see Equation (3)).

(7) $$\nabla \left(E\cdot E\right)=\left[\frac{\partial \left(E_{\mathrm{DC}}\cdot E_{\mathrm{DC}}\right)}{\partial r}\hat{r}+\frac{\partial \left(E_{\mathrm{DC}}\cdot E_{\mathrm{DC}}\right)}{r\partial \theta }\hat{\theta }+\frac{\partial \left(E_{\mathrm{DC}}\cdot E_{\mathrm{DC}}\right)}{\partial z}\hat{z}\right]\left(1+\alpha^{2}\right)=\frac{-2V_{0}{}^{2}\left(1+\alpha^{2}\right)}{\beta^{2}r^{3}}\hat{r}$$

Note that the θ and z components of the electric field gradients vanish due to the electric field being purely a function of the radial coordinate. In addition, note the contribution of the AC voltage through α stemming from the non-zero time-averaged nature of the square of the electric field. A spherical particle of diameter d flowing electrokinetically along an electric field line inside the microchannel (according to Equation (6)) experiences a dielectrophoretic force due to this electric field gradient. Using Equations (1) and (7), the force can be expressed as

(8) $$F_{\mathrm{DEP}}=\frac{-\pi }{2}d^{3}\varepsilon_{f}Real\left(f_{CM}\right)\frac{V_{0}{}^{2}\left(1+\alpha^{2}\right)}{\beta^{2}r^{3}}\hat{r}$$

Equation (8) shows that the negative DEP force is always directed along the positive radial direction (As $Real\left(f_{CM}\right)<0$, $F_{\mathrm{DEP}}>0$), and hence such a particle moves from the inner channel wall to the outer channel wall. Similarly, the positive DEP force is always directed along the negative radial direction (As $Real\left(f_{CM}\right)>0$, $F_{\mathrm{DEP}}<0$), and hence, such a particle moves from the outer channel wall to the inner channel wall. It is also clear from Equations (6) and (8) that the DEP force always acts on the particle in a direction orthogonal to its electrokinetic motion and hence cannot directly oppose the same. Hence, the curved arc microchannel is unable to immobilise i.e., trap particles dielectrophoretically, but can only allow a continuous radial deflection of the particles as they move inside the microchannel along the circumferential direction of the arc. In addition, a particle close to the microchannel walls, locally perturbs the electric field between itself and the wall, resulting in a wall repulsion force that always acts on the particle in a direction **n** normal to the wall (i.e., the radial direction in this case). Neglecting the surface conduction effects, the force $F_{w}$ acting on a particle sufficiently smaller than the channel curvature can be estimated up to a first order approximation as [50]

(9) $$F_{w}\approx \varepsilon_{f}\frac{d^{2}}{4}{\left(E_{\mathrm{DC}}{}^{W}\right)}^{2}\left(1+\alpha^{2}\right)f\left(\frac{d}{h}\right)\hat{n}\;\mathrm{where}\;f=\frac{3\pi }{256}{\left(\frac{d}{h}\right)}^{4}$$

where $E_{\mathrm{DC}}{}^{W}$ represents the scale of electric field near the wall, and *h* is the distance between the particle centre and the wall along the normal direction n pointing away from the wall. Similar to dielectrophoresis, the AC field contributes to the DC field in generating the repulsion effect because of the quadratic dependence on the electric field. Using this relation at the inner and outer walls of the circular arc microchannel, the wall repulsion forces at the inner (represented by the superscript “i”, i.e., $F_{w}{}^{i}$) and outer (represented by the superscript “o”, i.e., $F_{w}{}^{o}$) walls may be respectively approximated as [50]

(10) $$F_{w}{}^{i}\approx \frac{3{\pi \varepsilon }_{f}d^{6}{\left(E_{\mathrm{DC}}{}^{i}\right)}^{2}\left(1+\alpha^{2}\right)}{1024{\left(r-R_{i}\right)}^{4}}\hat{r};\;F_{w}{}^{o}\approx -\frac{3{\pi \varepsilon }_{f}d^{6}{\left(E_{\mathrm{DC}}{}^{o}\right)}^{2}\left(1+\alpha^{2}\right)}{1024{\left(R_{o}-r\right)}^{4}}\hat{r}$$

where $E_{\mathrm{DC}}{}^{i}$ and $E_{\mathrm{DC}}{}^{o}$ respectively indicate the electric field scales near the inner (i) and outer (o) walls of the microchannel and will be interpreted in more detail after a few steps. Note that the terms “$r-R_{i}$” and “$R_{o}-r$” represent the distance h between the particle centre-line and the inner and outer channel walls respectively, in a direction normal to the walls (i.e., the radial direction in this case). In addition, note that the negative sign for $F_{w}{}^{o}$ indicates that the action of the force is opposite to the sign convention of a positive radially outward unit vector $\hat{r}$.

The effective radial deflection force on the particle is obtained by the sum of Equations (8) and (10), which, after balancing against the Stokes drag force ($3{\pi \eta }_{f}{du}_{\mathrm{Def}}$) for a spherical particle, gives an expression for the terminal deflection velocity $u_{\mathrm{Def}}$ of the particle:

(11) $$u_{\mathrm{Def}}=\left[\frac{-d^{2}\varepsilon_{f}Real\left(f_{CM}\right)V_{0}{}^{2}\left(1+\alpha^{2}\right)}{6\beta^{2}r^{3}\eta_{f}}+\frac{\varepsilon_{f}d^{5}\left(1+\alpha^{2}\right)}{1024\eta_{f}}\left\{\frac{{\left(E_{\mathrm{DC}}{}^{i}\right)}^{2}}{{\left(r-R_{i}\right)}^{4}}-\frac{{\left(E_{\mathrm{DC}}{}^{o}\right)}^{2}}{{\left(R_{o}-r\right)}^{4}}\right\}\right]\hat{r}$$

Note that Equation (11) assumes a mass-less particle analysis, which holds for miniaturised systems owing to the negligible inertia of the micron-sized particles [18]. The time dependent position vector $r_{p}$ of the particle moving inside the microchannel can now be related to the particle velocity $u_{p}$ as

(12) $$u_{p}=u_{\mathrm{Def}}+u_{\mathrm{EK}}=\frac{dr_{p}}{\mathrm{dt}}=\frac{\mathrm{dr}}{\mathrm{dt}}\hat{r}+r\frac{d\theta }{\mathrm{dt}}\hat{\theta }+\frac{\mathrm{dz}}{\mathrm{dt}}\hat{z}$$

Recognising that the circumferential component of $u_{p}$ is represented entirely by the electrokinetic velocity $u_{\mathrm{EK}}$ (Equation (6)) and the radial component by the deflection velocity $u_{\mathrm{Def}}$ (Equation (11)), we can eliminate the vector notations and write

(13) $$\frac{\mathrm{dr}}{\mathrm{dt}}=\frac{-d^{2}\varepsilon_{f}Real\left(f_{CM}\right)V_{0}{}^{2}\left(1+\alpha^{2}\right)}{6\beta^{2}r^{3}\eta_{f}}+\frac{\varepsilon_{f}d^{5}\left(1+\alpha^{2}\right)}{1024\eta_{f}}\left\{\frac{{\left(E_{\mathrm{DC}}{}^{i}\right)}^{2}}{{\left(r-R_{i}\right)}^{4}}-\frac{{\left(E_{\mathrm{DC}}{}^{o}\right)}^{2}}{{\left(R_{o}-r\right)}^{4}}\right\};\;r\frac{d\theta }{\mathrm{dt}}=\frac{\varepsilon_{f}\left(\zeta_{p}-\zeta_{w}\right)V_{0}}{{\beta \eta }_{f}r}$$

which, after eliminating the time coordinate, gives

(14) $$\frac{\mathrm{dr}}{d\theta }=\frac{-d^{2}Real\left(f_{CM}\right)V_{0}\left(1+\alpha^{2}\right)}{6\beta r\left(\zeta_{p}-\zeta_{w}\right)}+\frac{\beta d^{5}\left(1+\alpha^{2}\right)}{1024\left(\zeta_{p}-\zeta_{w}\right)V_{0}}\left\{\frac{{\left(rE_{\mathrm{DC}}{}^{i}\right)}^{2}}{{\left(r-R_{i}\right)}^{4}}-\frac{{\left(rE_{\mathrm{DC}}{}^{o}\right)}^{2}}{{\left(R_{o}-r\right)}^{4}}\right\}$$

Note that the absence of electric field and its gradients in the z direction (i.e., along the channel depth) converts the 3D problem into a 2D problem in the r-θ plane. Equation (14) is an ordinary differential equation that represents the rate of change of the radial coordinate of the particle with respect to its circumferential coordinate and is therefore a direct indication of the dielectrophoretic deflection performance of the C-iDEP system. Note that the term $r^{2}$ arising after simplifying Equation (13) is taken inside the brackets with $E_{\mathrm{DC}}{}^{i}$ and $E_{\mathrm{DC}}{}^{o}$. For further analysis, it is assumed that the particle is sufficiently smaller than the geometry of the channel for the electric field scale around a particle at one wall to be unaffected by the other wall (i.e., for a semi-infinite domain approach to be valid). This assumption is reasonably good in practical microfluidic systems. Under this assumption, the local scales of the quantities $rE_{\mathrm{DC}}{}^{i}$ and $rE_{\mathrm{DC}}{}^{o}$ around the particle can be estimated using Equation (5) as rEDCi ~ Ri(V0βRi) ~ V0β and rEDCo ~ Ro(V0βRo) ~ V0β. Using these expressions, Equation (14) becomes

(15) $$\frac{\mathrm{dr}}{d\theta }=\frac{d^{2}V_{0}\left(1+\alpha^{2}\right)}{6\beta \left(\zeta_{p}-\zeta_{w}\right)}\left[\frac{-Real\left(f_{CM}\right)}{r}+\frac{3d^{3}}{512}\left\{\frac{1}{{\left(r-R_{i}\right)}^{4}}-\frac{1}{{\left(R_{o}-r\right)}^{4}}\right\}\right]$$

Equation (15) can be converted into a fully dimensionless equation by defining a dimensionless radial coordinate r^*^ and a dimensionless angular co-ordinate θ^*^ as $r^{*}=\left(r-R_{i}\right)/W$ and $\theta^{*}=\theta /\beta$ where $W=R_{o}-R_{i}$ is the channel width. In addition, the mean radius of curvature $R_{C}$ of the channel is introduced by using the relation $R_{C}=R_{i}+\left(W/2\right)$. With these substitutions, the non-dimensional form of Equation (15) can be written as

(16) $$\frac{{\mathrm{dr}}^{*}}{{d\theta }^{*}}=V_{App}{}^{*}d^{*}{}^{2}\left[\frac{-Real\left(f_{CM}\right)}{R_{C}{}^{*}+r^{*}-0.5}+\frac{3}{512}d^{*}{}^{3}\left\{\frac{1}{r^{*}{}^{4}}-\frac{1}{{\left(1-r^{*}\right)}^{4}}\right\}\right]$$

where

(17) $$V_{App}{}^{*}=\frac{V_{0}\left(1+\alpha^{2}\right)}{6\left(\zeta_{p}-\zeta_{w}\right)},R_{C}{}^{*}=\frac{R_{C}}{W},\;d^{*}=\frac{d}{W}$$

Equation (16) shows that the dielectrophoretic motion of a particle in a circular arc microchannel can be completely characterised using three dimensionless numbers, which are defined in Equation (17). $V_{App}{}^{*}$ is a measure of the strength of the applied voltage relative to the electrical double layer (EDL) potential, which the liquid induces at the surface of the particle and the channel wall. $V_{App}{}^{*}$ is positive for a counter-clockwise electrokinetic motion of particles and negative for a clockwise one. $R_{C}{}^{*}$ and $d^{*}$ respectively indicate the dimensionless curvature ratio of the microchannel and the dimensionless particle blockage ratio. It is also important to note that the particle motion is completely independent of the electrical permittivity and dynamic viscosity of the fluid because of the linear dependence of the individual particle velocity components on the quantity $\varepsilon_{f}/\eta_{f}$ and the resulting cancellation of this quantity while deriving the differential equation of the particle motion.

### 2.2. A Simplified Exact Solution

Equation (16) is not possible to be solved analytically using straightforward integration methods. However, it can be simplified to an elegant form if the influence of repulsion forces near the channel walls becomes negligible. This happens when the particle is treated as an infinitesimally small point, an assumption which holds if its size is much smaller compared to the characteristic dimensions of the channel geometry (in other words, $d^{*}\ll 1$ or d << W,$R_{C}$). In this scenario, Equation (16) becomes

(18) $$\frac{{\mathrm{dr}}^{*}}{{d\theta }^{*}}=\frac{-Real\left(f_{CM}\right)V_{App}{}^{*}d^{*}{}^{2}}{R_{C}{}^{*}+r^{*}-0.5}\;$$

The solution of Equation (18) between any two locations ($r_{1}{}^{*},\theta_{1}{}^{*}$) and ($r_{2}{}^{*},\theta_{2}{}^{*}$) in the channel can be derived as

(19) $$\frac{{\left(r_{2}{}^{*}+R_{C}{}^{*}-0.5\right)}^{2}-{\left(r_{1}{}^{*}+R_{C}{}^{*}-0.5\right)}^{2}}{2d^{*}{}^{2}Real\left(f_{CM}\right)}=V_{App}{}^{*}\left(\theta_{1}{}^{*}-\theta_{2}{}^{*}\right)$$

Equation (19) holds provided the particles are much smaller than the characteristic dimensions of the channel. This equation may also be viewed as an ideal solution for the particle motion inside the curved microchannel as it ignores the inevitable existence of wall repulsion forces in a special case.

Of particular importance for microfluidics engineers is the use of Equation (19) for quantifying the dimensionless design parameters in order to achieve a full focusing of an incoming particle suspension at the outlet of the channel. For example, in the case of a negative DEP occurring for a particle suspension moving electrokinetically in a positive angular (counter-clockwise) direction, full focusing can be realised by setting $r_{1}{}^{*}=0,\;r_{2}{}^{*}=1$ and $\theta_{1}{}^{*}=0,\;\theta_{2}{}^{*}=1$. Upon making these substitutions, one can simplify Equation (19) as

(20) $$\frac{R_{C}{}^{*}}{d^{*}{}^{2}Real\left(f_{CM}\right)}=-V_{App}{}^{*}$$

Note that even if the right hand side (RHS) of Equation (20) is negative because of $\theta_{1}{}^{*}<\theta_{2}{}^{*}$, the negative value of $Real\left(f_{CM}\right)$ maintains the positivity of the equation. It can be confirmed that Equation (20) retains its mathematical form for all other combinations of the directions of electrokinetic and DEP motion: (a) positive electrokinetic motion, positive DEP, (b) negative electrokinetic motion, negative DEP and (c) negative electrokinetic motion, positive DEP. The only change that occurs for each of these situations is the sign of the equation. Recognising this and taking the magnitude of $f_{CM}$ and ($\theta_{1}{}^{*}-\theta_{2}{}^{*}$) into account, the relationship between the dimensionless parameters for full focusing can be absorbed into an elegant expression as follows:

(21) $$\frac{\left|V_{App}{}^{*}\right|d^{*}{}^{2}\left|Real\left(f_{CM}\right)\right|}{R_{C}{}^{*}}=1$$

Equation (21) can be rearranged in several forms as shown below in order to determine the threshold value of a dimensionless design parameter for achieving a full focusing from the channel inlet to the channel outlet when the other parameters are known.

(22) $${\left|V_{App}{}^{*}\right|}_{\mathrm{Min}}=\frac{R_{C}{}^{*}}{d^{*}{}^{2}\left|Real\left(f_{CM}\right)\right|};\;d^{*}{}_{\mathrm{Min}}=\sqrt{\frac{R_{C}{}^{*}}{\left|V_{App}{}^{*}\right|\left|Real\left(f_{CM}\right)\right|}};\;R_{C}{{}^{*}}_{\mathrm{Max}}=\left|V_{App}{}^{*}\right|d^{*}{}^{2}\left|Real\left(f_{CM}\right)\right|$$

Equation (22), in combination with Equation (17), can be used to determine: (a) the minimum voltage that must be applied for fully focusing a given particle size inside a given channel geometry, (b) the smallest particle size that can be fully focused inside a given channel geometry due to a given applied voltage and (c) the maximum curvature radius which one can provide to the channel for fully focusing a given particle size due to a given applied voltage.

### 2.3. The Full Solution

Realistically, the particle size being finite and non-zero, the particle path-lines deviate from the ideal solution and the wall repulsion forces inevitably contribute to the dielectrophoretic focusing effects. Hence, the full solution of Equation (16) needs to be considered and critically evaluated for characterising the C-iDEP microchannel design. However, the integration methods for solving the equation analytically are not straightforward, and hence, it must be solved using a 1-D numerical integration. Considering the example of negative DEP and a positive electrokinetic motion as before, Equation (16) is integrated within the limits $r_{1}{}^{*},r_{2}{}^{*}$ and $\theta_{1}{}^{*},\theta_{2}{}^{*}$ for the radial and angular co-ordinates respectively. However, the finite size of the particle is now taken into account by setting the initial radial position, i.e., $r_{1}{}^{*}$ as $d^{*}/2$ instead of 0 (corresponding to the radial position Ri+d2). The angular coordinate limits are taken as 0 and 1 as before. Using these substitutions and rearranging Equation (16), we obtain

(23) $$\frac{1}{d^{*}{}^{2}}\overset{r_{2}{}^{*}}{\underset{\frac{d^{*}}{2}}{\int }}\frac{dr^{*}}{\left[\frac{-Real\left(f_{CM}\right)}{R_{C}{}^{*}+r^{*}-0.5}+\frac{3}{512}d^{*}{}^{3}\left\{\frac{1}{r^{*}{}^{4}}-\frac{1}{{\left(1-r^{*}\right)}^{4}}\right\}\right]}=-V_{App}{}^{*}$$

It must now be recognised that the full focusing of the particles will occur before they reach the outer channel wall, at an equilibrium radial co-ordinate $r_{Eq}{}^{*}$ where the DEP force is balanced by the wall repulsion force. The equilibrium co-ordinate can be obtained from Equation (16) by setting $dr^{*}/d\theta^{*}$ = 0 so that we get

(24) $$\frac{-Real\left(f_{CM}\right)}{R_{C}{}^{*}+r_{Eq}{}^{*}-0.5}+\frac{3}{512}d^{*}{}^{3}\left\{\frac{1}{r_{Eq}{{}^{*}}^{4}}-\frac{1}{{\left(1-r_{Eq}{}^{*}\right)}^{4}}\right\}=0$$

The solution of Equation (24) is then used as the integration limit for $r_{2}{}^{*}$ in Equation (23). It is important to note that a similar procedure is followed for all the other combinations of the directions of the DEP and electrokinetic motions, with the only difference being that the integration limit for $r_{1}{}^{*}$ in the case of positive DEP is defined as 1−(d*2) instead of 0 (corresponding to the radial position Ro−d2).

### 2.4. Data Analysis

Since the integration of the full solution is specific for a given combination of $Real\left(f_{CM}\right)$, $R_{C}{}^{*}$ and $d^{*}$, Equations (23) and (24) are numerically solved in MATLAB using the in-built integration functions for the following ranges of working parameters: (a) 15 values of $R_{C}{}^{*}$ ranging from 1 to 15 in intervals of 1, (b) 27 values of $d^{*}$, from 0.001 to 0.01 in steps of 0.001, from 0.01 to 0.02 in steps of 0.0025 and 0.02 to 0.15 in steps of 0.01 and (c) 10 values of $Real\left(f_{CM}\right)$ ranging from −0.05 to −0.5 in steps of −0.05 for negative DEP and from 0.1 to 1 in steps of 0.1 for positive DEP. This returns a total of 4050 $V_{App}{}^{*}$ values for each DEP direction and hence a total of 9100 data points. These parameter ranges are chosen in order to encompass the practical values of these parameters used in the published literature of curvature radii and the particle and cell sizes. A few example calculations of these are included in the Supplementary Materials S1.

For numerical integration purposes, the integration limit for the final position $r_{2}{}^{*}$ in Equation (23) is chosen to be 0.99$r_{Eq}{}^{*}$ for negative DEP and 1.01$r_{Eq}{}^{*}$ for positive DEP. This offset of 1% has to be chosen because Equation (24), which is also the denominator of the integrand in Equation (23), vanishes at $r_{2}{}^{*}=r_{Eq}{}^{*}$, thereby returning an indeterminate solution at that limit. As a result, the integration approaches the RHS of Equation (23) asymptotically (this is also confirmed from a trial and error study of the integration limit), and the offset is determined sufficient for tending to the solution it is theoretically expected to reach. 

It is found that the direction of the electrokinetic motion only changes the sign of the integration solution for both directions of DEP, which is expected as the deviation from the ideal solution, and the factors responsible for it exist in the radial direction alone. Hence, the magnitudes of $V_{App}{}^{*}$ and $f_{CM}$ are taken into account for the analysis, and only two sets of data points are generated, one for each direction of DEP. 

A visual inspection of the data points reveals that the value of $\left|V_{App}{}^{*}\right|$ varies linearly with $R_{C}{}^{*}$ and inversely with $d^{*}{}^{2}$ and $\left|Real\left(f_{CM}\right)\right|$ (which is also consistent with Equation (21)). Hence, curve fitting techniques are employed using the “Solver” function in Microsoft Excel to obtain a relationship between these parameters over the defined ranges of these parameters. As the particle blockage ratio $d^{*}$ is the dimensionless parameter solely responsible for the generation of the wall repulsion force and the resulting deviation from the ideal solution (Equation (21)), the generated data is rearranged to express a combination of $\left|V_{App}{}^{*}\right|,\;R_{C}{}^{*}$ and $\left|Real\left(f_{CM}\right)\right|$ in terms of $d^{*}$. First, Equation (21) is rewritten as [|VApp*||Real(fCM)|RC*]Exact=1d*2. Because the data is mathematically similar to this equation, it is processed to generate a three-parameter curve fit expressed by the following equations

(25) $${\left[\frac{\left|V_{App}{}^{*}\right|\left|Real\left(f_{CM}\right)\right|}{R_{C}{}^{*}}\right]}_{\mathrm{pDEP},\;\mathrm{Empirical}}=\frac{1}{d^{*}{}^{2}}-\frac{1.53}{d^{*}}+3.36$$

(26) $${\left[\frac{\left|V_{App}{}^{*}\right|\left|Real\left(f_{CM}\right)\right|}{R_{C}{}^{*}}\right]}_{\mathrm{nDEP},\;\mathrm{Empirical}}=\frac{1}{d^{*}{}^{2}}-\frac{2.49}{d^{*}}+6.75$$

where the curve fitting parameters are determined from the Solver function in Microsoft Excel by minimising the sum of % errors between the individual MATLAB data points and the corresponding curve fitting data points. The standard sum of least square minimisation technique is not used because the ultimate aim of this work is to analyse the deviation of the DEP particle dynamics from the simplified exact solution due to the inevitable existence of the wall repulsion force and to provide an equation to the scientific community which would be able to reasonably predict the particle dynamics for all the values of particle blockage ratios $d^{*}$ over the entire chosen parameter range. The sum of least squares method was attempted and was discarded because it was determined to significantly deviate from the MATLAB data for large values of $d^{*}$.

Minimising the sum of % errors generates the positive DEP (pDEP) curve fit equation (Equation (25)) with a mean % error of 1.31% and a standard deviation of 1.62%, with 194 outliers (which is about 5% of the total number of 4050 data points) deviating from the MATLAB data by more than 5%. Similarly, minimising the sum of % errors generates the negative DEP (nDEP) curve fit equation (Equation (26)) with a mean % error of 3.55% and a standard deviation of 3.25%, with 290 outliers (which is about 7% of the total number of 4050 data points) deviating from the MATLAB data by more than 8%. Although the nDEP data is a slightly poorer fit compared to the pDEP data, both the equations can be seen to overall provide a highly reliable prediction of the particle dynamics over the entire range of dimensionless parameters considered for this work. Supplementary Materials S2 and S3 provide the Excel files containing and confirming the aforementioned statistics for both positive and negative DEP. The data also justifies the reason for choosing the % error minimisation method over the sum of least squares method. 

The % deviation δ of the above curve fit equations from the simplified exact solution can then be calculated as

(27) $$\delta {\left(\%\right)}_{\mathrm{pDEP}}=100\left|\frac{\left({\left[\frac{\left|V_{App}{}^{*}\right|\left|f_{CM}\right|}{R_{C}{}^{*}}\right]}_{\mathrm{pDEP},\;\mathrm{Empirical}}-{\left[\frac{\left|V_{App}{}^{*}\right|\left|f_{CM}\right|}{R_{C}{}^{*}}\right]}_{\mathrm{Exact}}\right)}{{\left[\frac{\left|V_{App}{}^{*}\right|\left|f_{CM}\right|}{R_{C}{}^{*}}\right]}_{\mathrm{Exact}}}\right|=100\times \left|3.36d^{*}{}^{2}-1.53d^{*}\right|$$

(28) $$\delta {\left(\%\right)}_{\mathrm{nDEP}}=100\left|\frac{\left({\left[\frac{\left|V_{App}{}^{*}\right|\left|f_{CM}\right|}{R_{C}{}^{*}}\right]}_{\mathrm{nDEP},\;\mathrm{Empirical}}-{\left[\frac{\left|V_{App}{}^{*}\right|\left|f_{CM}\right|}{R_{C}{}^{*}}\right]}_{\mathrm{Exact}}\right)}{{\left[\frac{\left|V_{App}{}^{*}\right|\left|f_{CM}\right|}{R_{C}{}^{*}}\right]}_{\mathrm{Exact}}}\right|=100\times \left|6.75d^{*}{}^{2}-2.49d^{*}\right|$$

**Figure 2:**
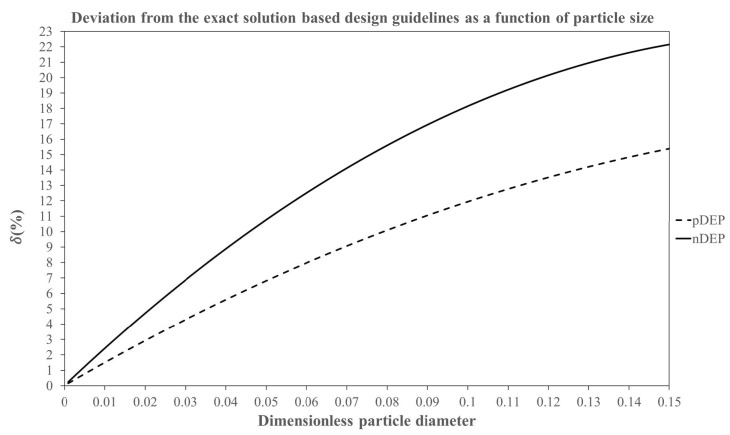
% Deviation of the dimensionless parameter $\left[\frac{\left|V_{App}{}^{*}\right|\left|Real\left(f_{CM}\right)\right|}{R_{C}{}^{*}}\right]$ generated from the curve fitting equations for pDEP (Equation (27)) and nDEP (Equation (28)) from its value predicted by the exact solution (Equation (21)) as a function of the dimensionless particle diameter $d^{*}$. Note that the curve fitting equations deviate increasingly from the exact solution for both pDEP and nDEP with increasing particle diameters, indicating the increasing influence of the wall repulsion forces on the C-iDEP particle dynamics with particle blockage.

The variation of δ as a function of $d^{*}$ for both pDEP and nDEP is shown in Figure 2. Figure 2 as well as Equations (27) and (28) show that as the particle blockage ratio increases, the deviation between the curve fit equation and the exact solution becomes larger, which is consistent with the underlying physics of the origins of the wall repulsion force contributions in $d^{*}$ and the resulting deviation of the particle path-line from the ideally expected path inside the microchannel. It is observed that for any given combination of design parameters, the data for negative DEP deviates more strongly from the ideal solution than positive DEP. This can be explained through the physics of C-iDEP systems and the wall repulsion forces as follows. Regardless of the direction of DEP forces, the influence of wall repulsion forces causes the particles to be fully focused over a smaller radial distance compared to the exact solution. The focusing of a particle undergoing negative DEP is assisted by the repulsion force at the inner wall and opposed by the repulsion at the outer wall. These repulsion forces interchange their functions for positive DEP. Since the inner wall repulsion force is stronger than the outer wall repulsion force (see Equation (10)), negative DEP focusing would require a smaller applied voltage and $\left|f_{CM}\right|$ or can tolerate a larger curvature than positive DEP for a given particle size. Hence, the value of |VApp*||Real(fCM)|RC* required for a full focusing of negative DEP would be smaller than positive DEP, leading to a greater deviation from the exact solution.

### 2.5. Numerical Model

COMSOL Multiphysics 5.3a, a commercial finite element code, was used for developing a dimensionless numerical model to validate the analytical solutions. A 2-D set up was sufficient for the analysis considering the absence of electric field along the thickness of the channel. A circular arc microchannel having a unit width, a mean radius of curvature of $R_{C}{}^{*}$(to be consistent with the normalisation definition of the mean curvature radius by the channel width) and a unit angle of 1 radian subtended at the centre of curvature was constructed. The dimensionless Laplace equation for electric potential, i.e., $\nabla^{*}{}^{2}V_{DC}{}^{*}=0$, was solved by defining a dimensionless gradient $\nabla^{*}$ (defined as $\nabla^{*}=W\nabla$) and a dimensionless DC electric potential $V_{DC}{}^{*}$ (defined as $V_{DC}{}^{*}=V/V_{0}$).

Equation (14) is also made non-dimensional through reference velocity scale $\frac{\varepsilon_{f}V_{0}\left(\zeta_{p}-\zeta_{w}\right)}{W\eta_{f}}$ and is expressed as follows upon substitution of the individual terms.

(29) $$u_{P}{}^{*}=E_{\mathrm{DC}}{}^{*}+\frac{V_{App}{}^{*}d^{*}{}^{2}}{2}\left[Real\left(f_{CM}\right)\nabla^{*}E_{\mathrm{DC}}{{}^{*}}^{2}+\frac{3}{256}d^{*}{}^{3}E_{\mathrm{DC}}{{}^{*}}^{2}\left\{\frac{1}{r^{*}{}^{4}}-\frac{1}{{\left(1-r^{*}\right)}^{4}}\right\}\hat{r}\right]$$

where $r^{*}$ is defined as before. The aforementioned unitless Laplace equation and Equation (29) were then solved using the “Electrostatics” interface and the particle tracing function of COMSOL Multiphysics respectively. As boundary conditions for the Laplace equation, Dirichlet boundary conditions for a unit DC voltage drop are applied across the circumferential direction (corresponding to the voltage drop of $V_{0}$ normalised in the dimensionless system), and the other channel walls are assumed electrically insulating. The dimensionless electric field, $E_{\mathrm{DC}}{}^{*}$, obtained from the finite element solution is then used in Equation (29) to calculate the particle path-lines. The 2D path-line images and the particle position data are generated using the COMSOL post-processing tools and exported for further analysis.

Two test particles that represent a stream of particles occupying a microchannel are chosen for the analysis. The particle whose dynamics are governed by the ideal solution is represented by a red colour in the results, and the particle whose dynamics are governed by the full solution is represented by the blue colour in the results. Considering the directions of the DEP forces, the starting point of the red (ideal) particle is chosen to be at $r^{*}=1,\;\theta^{*}=0$ for positive DEP and at $r^{*}=0,\;\theta^{*}=0$ for negative DEP. The starting point of the blue (realistic) particle, however, is chosen to be at the realistic co-ordinates (i.e., r*=1−d*2, θ*=0 for positive DEP and r*=d*2, θ*=0 for negative DEP) in order to consider the contributions of the repulsion forces. Upon choosing the aforementioned co-ordinates of the test particles, it is ensured that designing the microfluidic system for fully deflecting these particles would automatically ensure the maximum possible concentration of the stream of particles they represent.

A 2-D mapped mesh is used to generate a system of simultaneous finite element equations over the model geometries, and the equations are solved for each simulation in less than 10 s. 

## 3. Results and Discussions

As observed from the data shown in Figure 2, one can divide the working range of the particle blockage ratio into 3 regimes on the basis of the extent of deviation δ from the exact solution. The limits of these regimes were determined from a trial and error analysis of the path-lines of the aforementioned test particles, and the values of these limits were chosen purely based on a reasonable visual distinction between the ideal and the realistic particle path-lines. We acknowledge that this visual interpretation and hence the regime limits could vary slightly. However, we justify through our results that even for the highest particle size regime regardless of its limits, the ideal solution always assures a reliable design of the curved arc microchannel dielectrophoresis with a factor of safety and hence is ultimately proposed for the entire particle size range chosen. We do so using this section to fully analyse the utility of the exact solution and the empirical curve fit equations for each of the three regimes through an example case. Each case considers positive as well as negative DEP and compares the equation-based particle path-line predictions with numerically predicted results. For all the models, a microchannel subtending a unit angle of 1 radian in the centre and a curvature ratio of 5 is chosen. The magnitude of $Real\left(f_{CM}\right)$ is taken as 0.5 for the DEP forces.

### 3.1. Regime 1: δ≤5%

In this regime, the deviation between the curve fit equations and the exact solution is very small. Hence, the wall repulsion effect can be deemed negligible enough to affect the particle motion, and thus, the exact solution can be directly used for predicting the DEP particle dynamics in the microfluidic system. For pDEP systems, the data from Figure 2 show that this situation is applicable for particle blockage ratios, i.e., $d^{*}$ having values up to 0.0354. Similarly for nDEP systems, this situation applies for $d^{*}$ having values up to 0.0213. Since the exact solution can be applied for designing C-iDEP systems within this regime, it follows from Equation (21) that the value of the dimensionless parameter |VApp*||Real(fCM)|RC* cannot fall below 798 for pDEP and below 2204 for nDEP respectively, if the particle size $d^{*}$ to be dielectrophoretically focused falls below the aforementioned threshold values 

**Figure 3:**
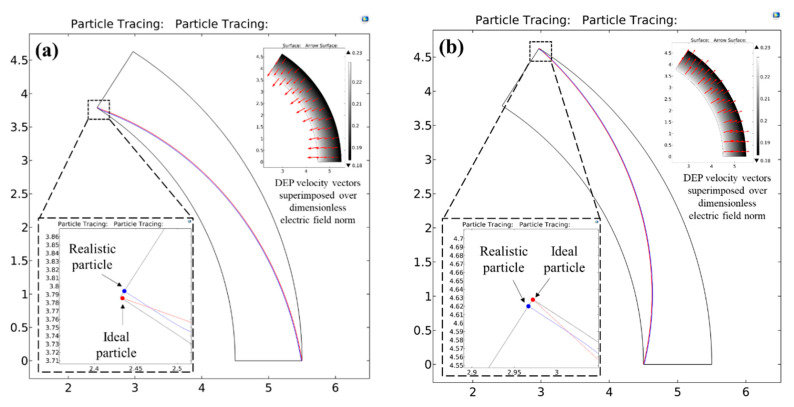
Numerically predicted path-lines for two test particles of a dimensionless diameter $d^{*}=0.005$ under the action of (**a**) positive DEP ($Real\left(f_{CM}\right)=0.5$) and (**b**) negative DEP ($Real\left(f_{CM}\right)=-0.5$) inside a circular arc microchannel having a curvature ratio $R_{C}{}^{*}$ of 5. The dimensionless applied voltage $\left|V_{App}{}^{*}\right|$ for both the DEP directions is 400,000, as calculated from the exact solution. The ideal particle is represented by the red colour and its motion is governed by the exact solution. The realistic particle is represented by the blue colour and its motion is governed by the full solution of particle motion inclusive of wall repulsion effects. This particle size demonstrates a regime 1 behaviour, where the design parameters for the C-iDEP microchannel can be determined reliably by the exact solution. The inset images show the DEP velocity vectors superimposed over the electric field norms, confirming an agreement of the simulated particle dynamics with their theoretical predictions.

Figure 3 shows an example situation of regime 1 with the help of two-dimensional path-lines of the two test particles (see Section 2.5) numerically predicted by COMSOL for a particle blockage ratio of 0.005 in both positive (Figure 3a) as well as negative DEP (Figure 3b) situations. The dimensionless applied voltage $\left|V_{App}{}^{*}\right|$ across the microchannel is chosen as 400,000 to maintain a consistency with the value of 40,000 for the dimensionless quantity |VApp*||Real(fCM)|RC* as per the exact solution.

As seen from the figures, the microchannel design parameters are able to fully concentrate the red as well as blue particles just at the outlet of the microchannel for both pDEP and nDEP, thereby confirming the applicability of the exact solution. As seen from the inset images, the DEP velocity vectors are directed from the inner channel wall to the outer channel wall for nDEP and vice versa for pDEP, which also confirms well with the theoretical predictions. Their increasing strength (indicated by the size of the arrows) in the region of high electric field also confirms the theoretical dependence of the DEP velocities on the radial position. A careful observation of the particle path-lines shows that the blue particle, as expected, is fully focused slightly before it reaches the destination channel walls and continues to travel along the equilibrium radial co-ordinate. This small discrepancy between the final radial coordinates of the red and blue particles can be explained through the curve fitting equations, which indicate that the value of |VApp*||Real(fCM)|RC* must be 39,697.36 and 39,508.75 for pDEP and nDEP respectively in order to ensure a full focusing of the blue particles at the channel outlet. Since both these values are smaller than the value predicted by the exact solution, the applied voltage is slightly stronger than what is required to fully focus these particles. However, for all practical purposes, it can be seen from the simulation that the % error between the final radial co-ordinates of both test particles is negligibly small, and hence, the exact solution holds in regime 1 for designing the C-iDEP systems.

One potential limitation of the utility of the analysis in this regime is that the values of $d^{*}$ in this regime are so small that the required voltages to focus these particles could induce strong Joule heating effects inside the channel which could cause the particle motion to deviate from the exact solution by virtue of change in the fluid properties and electrothermal effects. We expect that given the absence of abrupt and large changes in the electric field in C-iDEP systems, although the overall temperature itself could increase, the increase would be almost uniform in the r−θ plane (the small non-uniformities stemming from the depth-wise heat dissipation through typical Poly-dimethylsiloxane (PDMS)/glass-based fluidic systems [40]). Hence, the temperature gradients and the resulting electrothermal effects would still be small. Additionally, even if this temperature field would change the electrical permittivity and the dynamic viscosity of the liquid, we have demonstrated that the particle dynamics are independent of these two quantities, and hence, as a conclusion, we expect this analysis to work reliably for regime 1. However, we aim to still confirm our hypothesis experimentally in the near future.

In addition, it is important to note, particularly in context of this regime, that although C-iDEP systems are relatively safer from electrolysis risks compared to the conventional metallic microelectrode-based systems, the risk need not be eliminated. It can still become quite significant in this regime due to strong applied voltages and the subsequent electrokinetic flow of an electrolysis bubble into the microchannel to disrupt the particle dynamics. Microfluidics engineers and scientists can avoid this issue by using an inert electrode element like carbon. The use of this material has been well established in the microelectrode-based eDEP systems [51,52], but can be used for C-iDEP systems too without the loss of generality.

### 3.2. Regime 2: 5<δ≤12%

In this regime, the deviation between the curve fit equations and the exact solution is slightly too much larger than regime 1 for the effects of wall repulsion not to be neglected. This regime is characterised by particle blockage ratio $d^{*}$ ranging from 0.0354 to 0.1 for pDEP systems and from 0.0213 to 0.057 for nDEP systems. This corresponds to the quantity |VApp*||Real(fCM)|RC* falling within 88.06 and 758.12 for pDEP systems and 270.85 and 2094 for nDEP systems.

**Figure 4:**
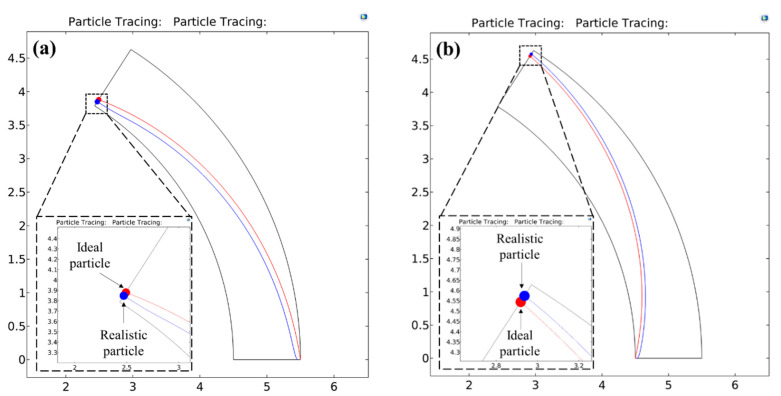
Numerically predicted path-lines for the ideal and realistic test particles under the action of (**a**) positive DEP ($Real\left(f_{CM}\right)=0.5$) and (**b**) negative DEP ($Real\left(f_{CM}\right)=-0.5$) inside a circular arc microchannel having a curvature ratio $R_{C}{}^{*}$ of 5. The dimensionless particle diameter $d^{*}$ is chosen as 0.08 for positive DEP and its motion is driven by a dimensionless voltage of $\left|V_{App}{}^{*}\right|=1404.85$. Similarly, the dimensionless particle diameter $d^{*}$ is chosen as 0.05 for negtive DEP and its motion is driven by a dimensionless voltage of $\left|V_{App}{}^{*}\right|=3569.5$. These particle sizes clearly demonstrate a regime 2 behaviour, where the design parameters for the C-iDEP microchannel can be determined reasonably by the exact solution but more reliably by the curve fitting equations.

Figure 4 shows example situations of regime 2 through a comparison of the numerically predicted path-lines of the two test particles. $d^{*}$ is taken as 0.08 for pDEP and 0.05 for nDEP. Substituting for these values in the curve fit equations (Equations (27) and (28)) gives the value of $\left|V_{App}{}^{*}\right|$ to be applied as 1404.85 for pDEP and 3569.5 for nDEP. As seen from Figure 4a,b, applying these respective voltages across the microchannel arc causes the blue particles to achieve a full deflection at the equilibrium radial co-ordinate of just around the microchannel outlet. However, it is observed that the red particles are not able to reach their destination channel wall and achieve a partial focusing for both pDEP and nDEP situations. From Equation (19), the theoretical radial co-ordinates that the red particle would achieve for pDEP and nDEP are 0.1107 and 0.901 respectively, which match very well with their numerically predicted counterparts of 0.111 and 0.9. This behaviour is consistent with the fact that the wall repulsion forces become more significant in this regime and are therefore able to focus the particles fully with the help of a smaller voltage than what is expected from the exact solution.

### 3.3. Regime 3: δ>12%

This regime applies for $d^{*}$ greater than 0.1 for pDEP and greater than 0.057 for nDEP. In this regime, the large values of $d^{*}$ result in highly significant contributions of the wall repulsion forces, and hence, the curve fit equations need to be evaluated for tracking the particle dynamics of the C-iDEP microdevice. Using the curve fit equations, it can be calculated that the regime 3 corresponds to a range of 88.06 or lower in the case of pDEP and 270.85 or lower in the case of nDEP for the dimensionless parameter |VApp*||Real(fCM)|RC* respectively. At the same time, using the range of $d^{*}$ for the exact solution returns a value of 100 or lower for pDEP and 307.78 or lower for nDEP. As reflected in the deviation δ, this value is large compared to the ranges defined by the curve fit equations, which implies that for given values of the curvature ratio, $R_{C}{}^{*}$, and $\left|Real\left(f_{CM}\right)\right|$, the applied voltage $\left|V_{App}{}^{*}\right|$ to achieve focusing using the exact solution would also be substantially larger than what is required realistically.

**Figure 5:**
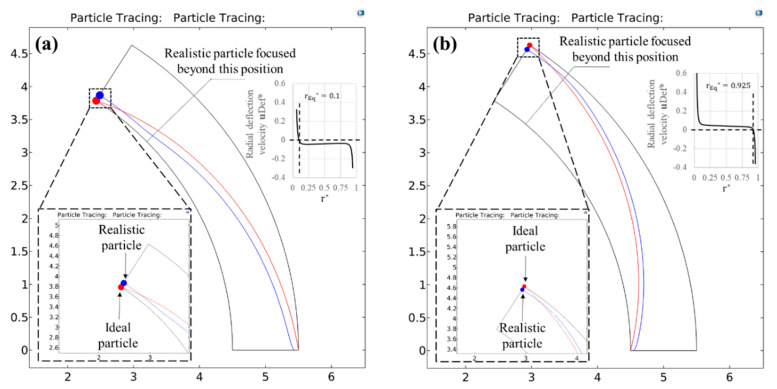
Numerically predicted path-lines for the ideal and realistic test particles under the action of (**a**) positive DEP ($Real\left(f_{CM}\right)=0.5$) and (**b**) negative DEP ($Real\left(f_{CM}\right)=-0.5$) inside a circular arc microchannel having a curvature ratio $R_{C}{}^{*}$ of 5. The dimensionless particle diameter $d^{*}$ is chosen as 0.12 for positive DEP and its motion is driven by a dimensionless voltage of $\left|V_{App}{}^{*}\right|=694.44$ predicted from the exact solution. Similarly, the dimensionless particle diameter $d^{*}$ is chosen as 0.08 for negative DEP and its motion is driven by a dimensionless voltage of $\left|V_{App}{}^{*}\right|=1562.5$ as predicted by the exact solution. These particle sizes demonstrate a regime 3 behaviour, where the realistic particle motion deviate significantly from the ideal behaviour. This is evident from the fact that the realistic particles in both cases of DEP are focused substantially before they reach the microchannel outlet. The location of full focusing along the arc length is highlighted by the dotted lines, where a kink in the particle path-line is visible, and the particle is seen traveling parallel to the channel wall beyond that point. This regime is characterised by more reduced voltage requirements to focus the particles fully due to the assistance of wall repulsion forces. The inset images represent a 1-D radial profile of the net radial particle velocity component, along with the identification of the equilibrium radial co-ordinates.

This behaviour is also confirmed from the numerically predicted path-lines of the test particles shown in Figure 5. The particle sizes, $d^{*}$, chosen are 0.12 and 0.08 for pDEP and nDEP respectively. Substituting these values in the exact solution returns a value of $\left|V_{App}{}^{*}\right|=$ 694.44 and 1562.5 for pDEP and nDEP respectively. As seen in the 2D figure, although this voltage fully deflects the red particle at the microchannel outlet for both pDEP and nDEP situations, the blue particle is seen to travel along the equilibrium radial co-ordinate for a considerable length of the arc. This is because the voltages applied are strong enough to fully focus the blue particles much before they reach the outlet. The inset images, which show the 1-D radial profiles of the dimensionless deflection velocity (DEP + wall repulsion), also support these observations qualitatively and quantitatively. The steep gradient of the velocities close to the wall stems from the strong wall repulsion forces that are dominant only at the walls for both the directions of DEP. The negative sign of the velocity on the profile indicates a motion opposite to the positive radial direction, i.e., from inner channel wall to the outer channel wall, so that the crossover point where the velocity switches sign is identified as the equilibrium co-ordinate. It is 0.1 for pDEP and 0.925 for nDEP, both of which agree very well with the theoretical predictions of 0.098 and 0.9244 respectively as per Equation (19).

All the results indicate that within the practical size ranges of cells and particles published in the existing literature, as $d^{*}$ increases and one transitions from regime 1 to regime 3, the voltage required for fully focusing the particles becomes increasingly smaller than what is predicted by the exact solution. Similarly, the curvature tolerance for obtaining the focusing also becomes larger. Since the design of experiments is always performed for safety, these results establish that using the exact solution and its corollaries (Equations (21) and (22)) as a guideline for designing the C-iDEP systems would guarantee a realistic focusing of the cells and particles with a factor of safety. Additionally, the actual experimental scenario often possesses uncertainties arising from factors like drift voltage or particle-particle interactions, which inevitably introduce deviations from the design parameter combination to be employed. In such a situation, having an equation which assures focusing before the particles reach the outlet would be a preferred choice for microfluidics engineers. If researchers still wish to have highly precise designs, Figure 2 can be used as a reference chart for back calculating the exact combination of design parameters as its utility has been confirmed through examples. 

In addition, one possible limitation of the realistic solutions, especially towards the upper limits of regime 3, is that with particle sizes becoming large, the volume they occupy within the channel can no longer be considered small enough for the point particle definition of DEP used in this work. Hence, aiming to use the curve fit equations for designing the systems in the upper limits of regime 3 or beyond would require the use of computationally expensive boundary element methods for accurate predictions. Using the exact solution as a design guideline would not only eliminate this issue but would also enable engineers to use a simple electronic calculator and design these systems.

### 3.4. Utility for Multiple Arcs

Since the arc microchannel forms the simplest design for C-iDEP systems, it would be of interest to explore the potential of the analysis of the particle path-line presented in this work for multiple channel turns by treating a single arc as a repeating unit. We consider a two-turn microchannel with identical arc geometries as an example for this work. Such designs are of great use if one needs to work with the same applied voltage as in the single turn and thereby reduce the voltage drop as well electric field strength per unit turn by a factor of two (identical turn geometries maintain a consistency with a single arc geometry and hence a 50% split). This reduction in the field strength would reduce the Joule heating and electrolysis risks associated with a single arc.

**Figure 6:**
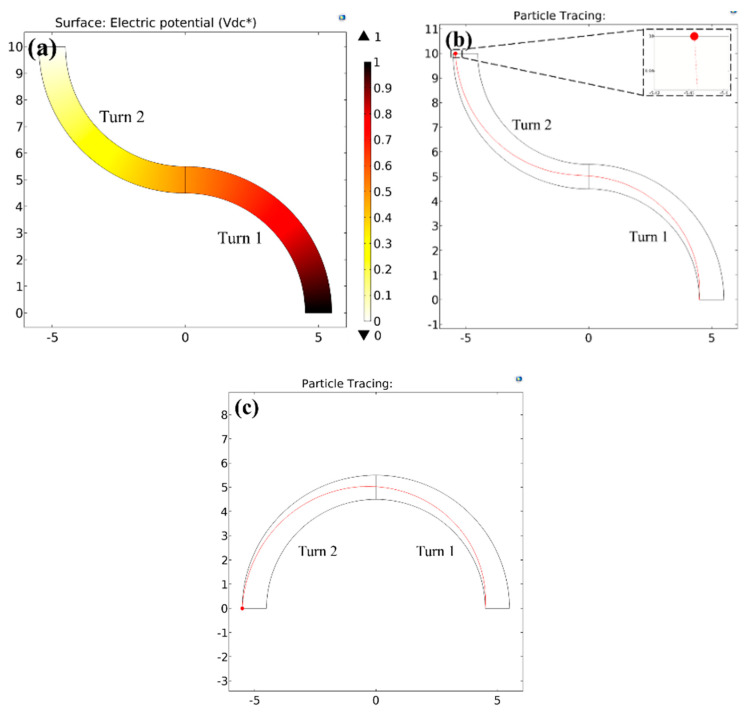
Numerically predicted behaviour of the negative DEP particle dynamics ($Real\left(f_{CM}\right)=-0.5$) inside a two-turn microchannel having a curvature ratio of $R_{C}{}^{*}$ of 5. (**a**) Numerically predicted dimensionless electric potential plot for a two-turn channel with opposing turns. (**b**) Path-lines of particles of diameter $d^{*}=0.02$ undergoing a DEP motion for a two-turn channel with opposing turns, under an applied voltage of $\left|V_{App}{}^{*}\right|=25,000$, with the inset showing the final position of the particle. (**c**) Path-lines of particles of diameter $d^{*}=0.02$ undergoing a DEP motion for a two-turn channel with unidirectional turns with other conditions identical to the opposing turn geometry.

Figure 6a shows a COMSOL simulation of the dimensionless voltage drop $V_{DC}{}^{*}$ for an example opposing two-turn microchannel (each arc being 90° with a curvature ratio of $R_{C}{}^{*}=5$) obtained by solving $\nabla^{*}{}^{2}V_{DC}{}^{*}=0$ (see Section 2.5), which confirms this hypothesis of 50% splitting of the voltage across each arc. 

In such a scenario, the analysis of particle position for a single arc can be theoretically used to calculate the final position of the particle at the end of the first turn, which is then used as an initial position for the next turn. We choose the exact solution for exploring this repeating unit approach owing to its established simplicity. Consider an example of a negative DEP ($Real\left(f_{CM}\right)=-0.5$) of an ideal particle of a dimensionless diameter $d^{*}$ of 0.02 (to be within the regime 1 where the exact solution applies) undergoing dielectrophoresis in the two-turn microchannel. In this situation, for a single turn arc, Equation (22) predicts that the voltage needed to fully deflect the particle to the opposite wall is $\left|V_{App}{}^{*}\right|=25,000$. For a two-turn microchannel, this voltage would be split across the two turns by a factor of two, leading to a voltage drop of 12,500 per turn. Setting $\left|V_{App}{}^{*}\right|=12,500$ for the initial radial position of a particle as the inner wall of the first arc ($r_{1}{}^{*}=0$) in Equation (19), one can calculate the final particle position at the end of the first turn as $r_{2}{{}^{*}}_{\mathrm{Turn}\;1}=0.5249$. 

Now if the two turns are unidirectional (i.e., in the same direction of curvature as in Figure 6c), this value becomes $r_{1}{{}^{*}}_{\mathrm{Turn}\;2}$ for the second turn. However, if the turns are opposing (i.e., change of curvature direction), this becomes ($1-r_{1}{{}^{*}}_{\mathrm{Turn}\;2}$) for the second turn. The values of $R_{C}{}^{*}$, $d^{*}$ and $\left|V_{App}{}^{*}\right|$ remain the same. Using Equation (19) again with these conditions for both cases of turns, we obtain $r_{2}{{}^{*}}_{\mathrm{Turn}\;2}=1$ for unidirectional turns and $r_{2}{{}^{*}}_{\mathrm{Turn}\;2}=0.9545$ for opposing turns. Interestingly, Figure 6b shows the final position of the particle at the end of the second opposing turn to be at $r_{2}{}^{*}=0.908$ (as seen from the inset, it is 5.408, which makes it 0.908 relative to 4.5, which is the radial co-ordinate of the inner wall of the second turn), indicating an offset of 0.0465 from the theoretical prediction. At the same time, Figure 6c shows an exact agreement with the theoretical prediction in unidirectional turns. This is not surprising because these essentially regenerate a single arc condition and the particle fully reaches the opposite wall.

It is clear from both these observations that the offset produced in the particle position for the opposing turn design, however small relative to the channel dimensions, comes from the change in the direction of the curvature in two-turn channels. Hence, although the analysis of the present work is quite consistent for two-turn designs, this offset points at the existence of an additional parameter or a condition that controls the particle motion in serpentine channels with two or multiple turns and hence can open up new opportunities for an extended analysis. In addition, the present form of the single arc equations in this work hold only if the channel width and curvature is constant. Hence, this research can also open up several directions for an extended analysis in designs where this need not be the case, such as spiral microchannels or curved microchannels with changing widths. Further, these analyses can be made more accurate by studying the wall repulsion force contribution to the particle motion, which would involve an extensive data analysis and numerical integrations. We aim to conduct these studies in the near future through follow-up works. However, we expect that regardless of the channel geometries discussed above, the repulsion forces would still be consistent in their physics and allow particle manipulation over a smaller arc length compared to the exact solution so that the exact solution would remain a preferred design choice.

## 4. Conclusions and Future Work 

In this work, we have provided comprehensive theoretical guidelines for designing curvature-induced dielectrophoresis-based particle concentration systems under the action of both DC and DC biased AC voltages and confirmed them extensively using two-dimensional finite element simulations. An arc microchannel, which forms the most basic design for such systems, is used to provide a detailed mathematical treatment. We have derived an elegant exact solution from first principles which shows that the full focusing of a given size of particles within a given geometry of microchannel is completely governed by three dimensionless parameters and the Clausius-Mossotti factor. These are the applied voltage strength relative to the wall-particle zeta potentials, the curvature ratio and the particle blockage ratio or dimensionless particle diameter. 

It is also shown with computational validations that wall repulsion forces which exist at the channel wall are strong functions of the particle blockage ratio alone and perturb the particle dynamics from those predicted by the exact solution. Based on this observation, an extensive numerical integration and test particle analysis is performed over a wide range of practically relevant values of dimensionless parameters to generate curve fitting equations that mutually relate the parameters to each other. Based on the extent of deviation this data exhibits from the exact solutions, three regimes are identified according to a specific range of particle sizes. An example situation from each regime is considered and then the particle path-lines predicted using COMSOL are compared with the theoretically predicted values with quantitative agreement with exact as well as curve fit equations, thereby establishing the reliability of both the equations. The results fully justify the utility of the exact solution to design the arc microchannels with a factor of safety over the entire working range of particles chosen, thereby establishing a calculator-based tool for microfluidics engineers interested in designing this system.

As introduced before in the theory, the present work is applicable for several potential uses in cell research as illustrated ahead. (a) For instance, for cells with well characterised dielectric properties, the analytical model can be used to design a simple arc microchannel, which, either through a positive or a negative DEP, would generate a narrow, highly-concentrated stream of single cells (like the coaches of a train) which can then be directed into further downstream lab-on-a-chip operations such as cell counters, cell culturing chambers and sensors. (b) The analytical equations can also be potentially used for obtaining useful information about the properties of uncharacterised cells in combination with the multi-shell model. (c) The analytical equation can also be used to obtain a size-based separation of cells from a binary mixture by exploiting the wall repulsion forces, which would focus them at different equilibrium coordinates [44].

Apart from a direct use for single arc microchannel design, this work is also shown to open up several new directions for establishing theoretical guidelines to design more complex C-iDEP-based systems involving a change in the strength and/or the direction of curvature. These are ongoing explorations as part of our research and are expected to generate comprehensive theoretical bases for these designs in near future. Establishing experimental protocols to support the dimensionless analysis and to investigate Joule heating effects in these devices is also under consideration.

## Supplementary Materials

The following are available online at https://www.mdpi.com/2072-666X/11/7/707/s1. S1: Provides calculations for a few example biological particles and cells suspended in the curved microchannels having the aforementioned range of widths; S2: nDEP_3 parameter fit; S3: pDEP2_3 parameter fit.
