# Supplementary material for: Analytical Guidelines for Designing Curvature-Induced Dielectrophoretic Particle Manipulation Systems
Source: Micromachines (Basel). 2020 Jul 21;11(7):707. doi: 10.3390/mi11070707 (PMC7407939; doi:10.3390/mi11070707)
Supplement: Supplementary file 1 [file micromachines-11-00707-s001.zip › micromachines-861267-supplementary-for publish.docx]

Supplementary information

Analytical Guidelines for Designing Curvature-Induced Dielectrophoretic Particle Manipulation Systems

Akshay Kale, Amirreza Malekanfard and Xiangchun Xuan

S1. Example calculations for practically relevant dimensionless parameters

The published literature of C-iDEP systems uses curved microchannels with widths ranging from 50 µm to 200 µm and the curvature radius from 300 µm to 500 µm. Using equation 17, the practically relevant range of the dimensionless curvature ratio can be calculated from the definition as,

| ${{R_{C}}^{*}}_{\mathrm{Min}}=\frac{300 \mu m}{200 \mu m}=1.5; {{R_{C}}^{*}}_{\mathrm{Max}}=\frac{500 \mu m}{50 \mu m}=10$ | (S1.1) |
| --- | --- |

Thus, it can be confirmed that the range of curvature ratios chosen for the analysis completely encompasses the practically relevant values

**Table S1.** Provides calculations for a few example biological particles and cells suspended in the curved microchannels having the aforementioned range of widths.

| **Biological Particle Type.** | **Mean Diameter d (µm)** | ${\mathbf{d}^{\boldsymbol{*}}}_{\mathbf{Min}}\boldsymbol{=}\frac{\mathbf{d}}{\mathbf{W}_{\mathbf{Max}}}$ | ${\mathbf{d}^{\boldsymbol{*}}}_{\mathbf{Max}}\boldsymbol{=}\frac{\mathbf{d}}{\mathbf{W}_{\mathbf{Min}}}$ |
| --- | --- | --- | --- |
| Red blood cell (RBC) | 6 µm | 0.03 | 0.12 |
| Algae (*C. Reindharti*) | 10 µm | 0.05 | 0.2 |
| DNA | 2.5 nm | < 0.0001 | < 0.0001 |
| *E. Coli* | 0.25 µm – 1 µm | 0.005 | 0.02 |
| Human Mesenchymal Stem cells (HMSC) | 15 µm | 0.075 | 0.3 |
| Yeast cells | 3 – 4 µm | 0.02 | 0.08 |

Table 1. Calculation of the dimensionless particle blockage ratio $d^{*}$ for practically relevant bioparticle suspensions in C-iDEP systems .

Table S1 shows that the range of the practically relevant particle blockage ratios $d^{*}$ is almost fully encompassed by the chosen range for this work. While the limits for Human mesenchymal stem cells and Algae cells fall slightly outside this range, the nature of the exact solution to provide a design for safety applies automatically for these values too.

| 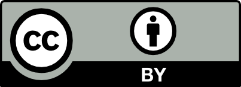 | © 2020 by the authors. Submitted for possible open access publication under the terms and conditions of the Creative Commons Attribution (CC BY) license (http://creativecommons.org/licenses/by/4.0/). |
| --- | --- |
